# Social Complexity and Nesting Habits Are Factors in the Evolution of Antimicrobial Defences in Wasps

**DOI:** 10.1371/journal.pone.0021763

**Published:** 2011-07-06

**Authors:** Stephen J. Hoggard, Peter D. Wilson, Andrew J. Beattie, Adam J. Stow

**Affiliations:** Department of Biological Sciences, Macquarie University, North Ryde, Australia; British Columbia Centre for Excellence in HIV/AIDS, Canada

## Abstract

Microbial diseases are important selective agents in social insects and one major defense mechanism is the secretion of cuticular antimicrobial compounds. We hypothesized that given differences in group size, social complexity, and nest type the secretions of these antimicrobials will be under different selective pressures. To test this we extracted secretions from nine wasp species of varying social complexity and nesting habits and assayed their antimicrobial compounds against cultures of *Staphylococcus aureus*. These data were then combined with phylogenetic data to provide an evolutionary context. Social species showed significantly higher (18x) antimicrobial activity than solitary species and species with paper nests showed significantly higher (11x) antimicrobial activity than those which excavated burrows. Mud-nest species showed no antimicrobial activity. Solitary, burrow-provisioning wasps diverged at more basal nodes of the phylogenetic trees, while social wasps diverged from the most recent nodes. These data suggest that antimicrobial defences may have evolved in response to ground-dwelling pathogens but the most important variable leading to increased antimicrobial strength was increase in group size and social complexity.

## Introduction

Disease risk in social insects can be influenced by a variety of intrinsic and extrinsic factors. Large group sizes and limited genetic diversity commonly associated with social species are known to be factors [1] and different nest substrates and climates present arrays of pathogens which may compromise a colony. Despite this, social Hymenoptera have successfully established on every continent (except Antarctica) suggesting that they have found one or more strategies to successfully combat disease. Indeed, multiple disease resistance strategies have been observed in various hymenopteran species including behavioural (e.g. allogrooming, nest cleaning), genetic (e.g. increased diversity) or biochemical (e.g. immune response, antibiotic secretions) [2], [3].

Cuticular antimicrobial compounds [1], [4], [5] are important because they target pathogens before they can infect an individual. Previous studies have shown that antimicrobial activity scales positively with group size in both bees and thrips [1], [4] supporting the theory that larger group sizes lead to greater risk of disease. However, in order to establish the generality of these findings, more insect lineages should be examined.

Within the Hymenoptera, wasps are of particular interest as both bees and ants arose from wasp lineages (Apoidea and Vespoidea superfamilies respectively [6]) and examples of all major nesting habits and levels social complexity are found within extant wasp taxa [7]. Additionally, antibacterial peptides have been isolated from the cuticle and venom of social wasp species [5]. Therefore, wasps provide the opportunity to examine the factors affecting disease resistance from some of the most primitive states up until the most recent and complex derivatives.

Given the diversity of wasp taxa we predicted that we would observe a large amount of variation in the relative strength of antimicrobial defences among species, based on life history traits. For this study, we extracted putative antimicrobial compounds from the cuticle of a variety of wasp species which span a range of nesting habits and social complexity. Using an established bioassay [8], the activity of these compounds was measured and compared. Based on previous studies we expected those species with the greatest group sizes (i.e. the social species) to possess the strongest antimicrobial compounds.

According to published phylogenies [6], [9] solitary wasp species are ancestral to social wasp lineages. Individuals of solitary species do not cooperate with one another and aggregations, if any, result from the availability of limited nesting sites [10], [11]. While not subjected to the evolutionary pressures of disease arising from sociality, as the ancestral lineage of social species, we predicted that solitary wasps would possess weak cuticular antimicrobial compounds. In contrast, for social species we predicted they would possess strong cuticular antimicrobial compounds used to protect themselves from disease risks associated with large, high-density group sizes. To test this hypothesis, we performed a phylogenetic reconstruction of the examined species as there are no comprehensive phylogenies for Australian vespid or apoid wasps. These data provided our results with an evolutionary context, allowing for inferences to be made regarding the order in which traits evolved or co-evolved.

## Materials and Methods

### Ethics Statement

No animal ethics approval was required for this study however wasps were incapacitated with carbon dioxide during sampling and prior to extraction of cuticular compounds and internal tissue to minimise stress to the animals.

### Sampling locations and species identification

1268 individual wasps comprising members of nine different species were collected from public land across Sydney (New South Wales) and Alice Springs (Northern Territory), Australia. Species were identified by morphology and sequencing of 28S nrDNA and COI mtDNA fragments (see below). In four wasp species, identification could only be resolved to genus-level due to lack of identification keys for many Australian wasp genera. In one instance, identification could only be resolved to the level of sub-family (designated as ‘Pepsinae Sp1’). Species were then categorised by social complexity (social, communal aggregator or solitary) and by nest type (paper nest, mud nest or burrow; Table 1), representing broad categories of group size and environmental exposure. Paper nests were defined as clusters of cells constructed from pulp and attached to the substrate by a petiole [7]. Mud nests were defined as sealed and provisioned cells constructed from mud and above-ground [12], [13]. Burrows were defined as either provisioned terrestrial burrows [10], [14], or as those species which burrowed for prey which was subsequently paralysed and ectoparasitised [15].

**Table 1:** Characterisation of wasp species.

Species (Family)	*n*	Sociality	Nest type	IC50 (±95% CI)	*n_r_*
*Polistes humilis* (Vespidae)	1077 (10)	Soc.	Paper	6.03 (±2.26)	28
*Ropalidia plebeiana* (Vespidae)	49 (2)	Soc.	Paper	7.58 (±5.91)	5
*Bembix sp.* (Crabronidae)	83	Com	Burrow	31.97 (±27.62)	6
*Austroscolia sp.* (Scoliidae)	47	Sol.	Burrow	158.27 (±152.82)	5 (3)*
*Cryptocheilus sp.* (Pompilidae)	4	Sol.	Burrow	14.47	1
Pepsinae Sp1 (Pompilidae)	1	Sol.	Burrow	90.26	1
*Abispa ephippium* (Vespidae)	1	Sol.	Mud	No Inhibition	1
*Sceliphron laetum* (Sphecidae)	5	Sol.	Mud	No Inhibition	2
*Delta sp.* (Vespidae)	1	Sol.	Mud	No Inhibition	1

*n*: number of individuals (number of colonies for social species); Sociality: social (Soc.), communal aggregator (Com.), solitary (Sol.); IC50: mean equivalent surface area (mm^2^) of wasp cuticle required to kill or inhibit 50% of *S. aureus* growth; *n_r_*: number of replicates per species.

*Only three replicates for *Austroscolia sp.* showed activity over the assayed concentration gradient and the IC50 value given was calculated using only these data.

### Bioassay

Putative antimicrobial compounds were assayed by established methods to assay antimicrobial compounds obtained from thrips and bees [1], [4] and were removed from the cuticle of live wasps by washing whole animals with 70% ethanol for 10 minutes, followed by two rinses to maximise extraction. Solvents were removed by vacuum evaporated at 25°C and the recovered residue was resuspended in LB broth. Extracts were assayed against *Staphylococcus aureus* using opposing gradients of extract concentration and cell numbers across rows of 12 wells in 96-well microtitre plates [8]. As *S. aureus* is exclusively found in birds and mammals [16], it is unlikely that any species of wasp has developed pathogen-specific defensive compounds that would bias our study of broad-scale antimicrobial compounds. Concentration-growth curves were generated for each species with a minimum of five replicates per species, and a minimum of three replicates per colony for social species, when sample size permitted. Three control rows were used in each assay: LB broth, resuspended extract with LB broth and a gradient of *S. aureus* cell numbers with LB broth. Initially, the maximum concentration of extract used was equivalent to a single wasp, however, preliminary assays indicated this was too low to detect activity in many species. Where additional samples were available the maximum concentration of extract was increased to 2.0 (*Cryptocheilus sp.*, *Sceliphron laetum* and *Austroscolia sp.*) or 4.0 (*Bembix sp.*). Where only a single individual was collected (*Delta sp.*, *Abispa ephippium* and Pepsinae Sp1) the highest concentration of extract was equivalent to 0.5 wasps. For social and communal aggregator species washes from between three and eight individuals were pooled and then diluted to the required wasp equivalent concentrations. All assays used a one-half serial dilution for both the *S. aureus* and extract gradients. Following incubation at 37°C for 19 h, growth in treatment and control wells was measured as an increase in optical density (OD) at 590 nm. These data were expressed as [increase in OD of treatment well]/[increase in OD of control well] and then used to determine the concentration of extract required to kill or inhibit 50% of *S. aureus* growth (herein referred to as IC50). A total of 50 assays were performed across the nine species (Table 1).

### Calculating Relative Antimicrobial Strength

Following Smith *et al.* (2008), a modified Gompertz function was fitted to the data using R (version 2.5.1 [17]) to calculate the IC50 value for each assay. Mean IC50 (±95% CI) was calculated for each species and each category of social complexity and nest type. Two-sample t-tests were performed between pairs of social complexity and nest type categories. To standardise measurements across different species, concentrations of wasp equivalents were converted to concentrations of equivalent surface area. Adapting methods previously applied to bees [1], mean surface area for each species was estimated by generating elliptical cylinders using measurements from up to ten individuals per species. Although regular cylinders would have been appropriate for most species, some of the larger species exhibited up to ∼1.5x difference between height and width, rendering regular cylinders an imprecise measure.

### DNA Extraction and Amplification

Using one member of each sampled species, DNA was extracted from internal tissues in the thoracic region of wasps using proteinase-K and ‘salting-out’ [18]. Phylogenetic reconstruction was performed using two gene fragments; 28S nuclear rDNA and COI mitochondrial DNA. Amplification of the 28S gene fragments was performed using primers previously used in the construction of microgastrid wasp phylogenies [19] and COI gene fragments amplifications were performed using generic invertebrate primers for that region [20]. PCRs for both gene fragment were carried out in 10 µL volumes containing 0.5U of GoTaq Flexi DNA polymerase (Promega), 1 µM forward primer, 1 µM reverse primer, 0.8 µM DNTPs, 1x GoTaq Buffer (Promega) and 2.0 mM MgCl_2_. PCR amplifications had an initial denaturation at 94°C for 3 min followed by six ‘touch down’ cycles of 94°C denaturation for 30 s, annealing temperatures (60°C, 58°C, 56°C, 54°C, 52°C, 50°C) for 30 s and an extension step of 72°C for 45 s. On the completion of the last touchdown cycle, another 35 cycles were carried out at 50°C annealing temperature and a final extension of 10 min at 72°C. Following PCR amplicons were purified using ExoSap-IT (USB) according to the manufacturer instructions and purified products were sequenced using their corresponding forward primers with dye terminator reactions on a 3130x1 Genetic Analyser (Applied Biosystems).

### Sequence Alignment

Phylogenetic reconstruction of nine wasp species (plus *Apis mellifera* as an outgroup) was performed using a 941 bp sequence generated by concatenating the two gene fragments: 28S nrDNA (484 bp) and COI mtDNA (457 bp). Generated sequences (Table 2) plus corresponding sequences from *A. mellifera* acquired from GenBank (28S: AJ302936.1; COI: FJ582092.1) were aligned using the ClustalW option with default parameters in MEGA v4.0 [21]. Length polymorphisms in sequence data were removed following alignment and prior to concatenation of gene fragments. Concatenation of the two sequences was deemed appropriate as a partition homogeneity test performed in PAUP* v4.0b10 [22] revealed no significant incongruencies between the two data sets (p = 0.124). Phylogenies were created using both distance-based (neighbour-joining) and Bayesian methods (maximum clade credibility).

**Table 2:** GenBank accession numbers by species.

Species name	28S	COI
*Polistes humilis*	JF510015	JF510006
*Ropalidia plebeiana*	JF510016	JF510007
*Bembix sp.*	JF510020	JF510011
*Austroscolia sp.*	JF510021	JF510012
*Cryptocheilus sp.*	JF510022	JF510013
Pepsinae Sp1	JF510023	JF510014
*Abispa ephippium*	JF510017	JF510008
*Sceliphron laetum*	JF510019	JF510010
*Delta sp.*	JF510018	JF510009

28S: GenBank accession number for the amplified 28S nrDNA fragment sequence; COI: GenBank accession number for the amplified COI mtDNA fragment sequence.

### Distance-based phylogenetic analysis

Neighbour-joining phylogenetic reconstruction was performed in MEGA v4.0 [21] using the maximum composite likelihood model. Both transition and transversion substitutions were included, assuming homogeneous patterns among lineages and uniform rates among sites. Gaps were treated as complete deletions. Bootstrap values were obtained using 10000 replicates.

### Bayesian phylogenetic analysis

Bayesian phylogenetic analysis was performed using BEAST v1.5.4 [23]. The input file for BEAST was generated using BEAUti v1.5.4. Trees were generated using a single MCMC chain of 20 million steps sampling every 5000 steps. Generalised time-reversible plus gamma (GTR+G) was selected as the model for nucleotide substitution using jModelTest v0.1.1 [24]. The molecular clock rate was fixed to 1.0 and the Yule process was selected as the tree prior. All other parameters were left in their default state as generated by BEAUti. Integrity of generated data was checked using Tracer v1.5 [25]. TreeAnnotator v1.5.4 (distributed with BEAST v.1.5.4) was used to generate a maximum clade credibility tree (MCC) using a burn-in period of 400 trees and a posterior probability limit of 0.5. The MCC tree was visualised using FigTree v1.3.1 [26].

## Results

### Antimicrobial Activity

IC50 values could be calculated for 44 of the 50 assays performed (Table 1). No antimicrobial activity was observed from species belonging to the ‘mud nest’ category. Two of the five assays performed using extract obtained from *Austroscolia sp.* also showed no activity hence IC50 values were calculated using data from the three assays for which IC50 values could be calculated. When grouped by social complexity, mean IC50 values for social species were significantly lower than those of solitary species (Two-sample t-test: social = 6.26, solitary = 115.93; p = 0.038). When grouped by nest type, mean IC50 values for paper nest species were significantly lower than those of burrow species (Two-sample t-test: paper nest = 6.26, burrow = 70.13; p = 0.015). As no species belonging to the ‘mud nest’ category showed any antimicrobial activity it was not possible to compare mean IC50 values with the other nest types. Similarly these species were not included when calculating differences in mean IC50 by social complexity.

### Phylogenetic Reconstruction

**Figure 1:**
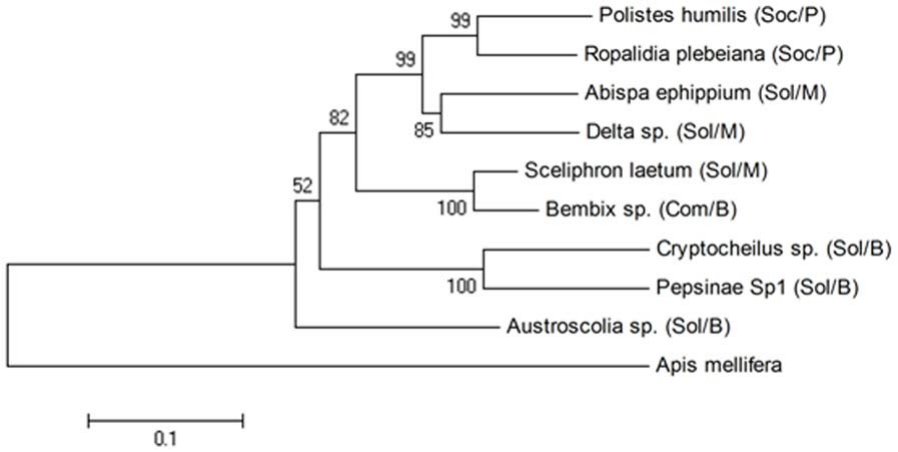
Distance-based neighbour-joining tree. Neighbour-joining phylogenetic reconstruction of nine wasp species using 941 bp sequence generated by concatenating the two gene fragments: 28S nrDNA (484 bp) and COI mtDNA (457 bp). Social complexity and nest type are indicated after the species names; social (Sol), communal aggregator (Com), solitary (Soc), paper nest (P), mud nest (M) and burrower (B). Bootstrap values were obtained using 10000 replicates.

Following the removal of length polymorphisms and concatenation of 28S and COI gene fragments, of the 941 bp sequence, 651 bp were found to be variable of which 346 bp were parsimony informative. Both the neighbour-joining and maximum clade credibility trees were highly congruent, except when placing the clades containing the two pompilid species and *Austroscolia sp.* which were switched between the two trees. In both trees the placement of *Austroscolia sp.* was the least supported branch (bootstrap support of 0.52; credibility support of 0.7853). Additionally, our phylogenetic reconstruction showed that solitary burrowing wasps diverged at basal nodes in both trees and that social lineages arose following a single divergence event (bootstrap support of 0.82; credibility support of 1.0; Figure 1).

## Discussion

It is clear from the data that levels of antimicrobial defences vary among different wasp taxa and that these differences are strongly linked to levels of social complexity. When examined together, the phylogenetic and antimicrobial data suggest that the production of antimicrobial compounds may have first arisen in solitary wasps as a response to environmental, probably soil-borne, pathogens.

### Social complexity

We observed an eighteen-fold difference in the mean strength of cuticular compounds when comparing social and solitary wasp species. This difference did not take into account those species for which a mean value could not be calculated, thus the actual difference between the two groups could be much higher. This finding is consistent with previous studies linking increased antimicrobial defences with group size and sociality [1], [4]. As wasps are an ancestral lineage in Hymenoptera [6], [9] and this pattern has already been demonstrated among bees [1] the relationship may hold throughout the social Hymenoptera.

### Nest type

Burrowing wasps, exposed to soil-based pathogens, may have developed broad-scale antimicrobial defences in response. These may have evolved into stronger compounds in the social lineages. Perhaps the lack of antimicrobial activity in solitary species that construct mud nests is because they are constructed above-ground where there is less risk of disease. Alternatively, individual species may have developed specific compounds to combat niche pathogens which are ineffective against *S. aureus*. We acknowledge that there may be confounding of results when examining comparisons of sociality and nest types as both ‘social’ species were also ‘paper nest’ species. With a sufficiently large sample size it may be possible to separate the effects of each of these traits, however time required to perform a single assay prohibits this.

### Phylogenetic considerations

Traditionally, wasp lineages have been placed in one of three distinct superfamilies (Vespoidea, Apoidea and Chrysidoidea) [6] however evidence from recent molecular-based phylogenies [9] has cast doubt on traditional taxonomies [9] which may explain some of the incongruencies between the phylogenetic trees presented in this paper and pre-existing, morphology-based phylogenies [6]. Our placement of species belonging to Vespidae is supported by both traditional taxonomic and more recent molecular phylogenies [6], [27], however our placement of *Sceliphron* and *Bembix* (which belong to the Apoidea superfamily) within Vespoidea is more congruent with molecular phylogenies published by Pilgrim *et al.* [9]. This is unsurprising as we did not use morphological data in our phylogenetic reconstruction, however to ensure that this result was not due to outgroup choice, we replicated our phylogenetic analysis replacing *Apis mellifera* (Apoidea) with *Chrysis cembricola* (Chrysididae; 28S: GQ374718.1; COI: GQ374633.1) as an outgroup (Chrysidoidea is a sister taxa to both Apoidea and Vespoidea). This substitution did not change the placement of Apoidea species or branch support for their nodes. We similarly removed *Apis mellifera* without replacement and again, this did not alter the placement of Apoidea species within the trees relative to the other taxa. The only difference observed between any of these trees (excepting minor changes to branch support values) was the placement of *Austroscolia* and Pompilidae taxa, which as previously stated, are the least supported branches in the analysis.

This study provides evidence for the origin of antimicrobial defences in wasps and Hymenoptera as a whole, and increases our understanding of trends in disease resistance strategies in all social insects. Assaying against pathogens for which wasps have no evolutionary relationship revealed those species which have potentially evolved to cope with wide-ranging or rapidly evolving pathogenic threats. The absence of such a response in mud-nest constructing species may be indicate that they are not subject to the same pathogenic evolutionary constraints as social and ground-dwelling wasp species. Further investigation is required to determine whether these species have lost their antimicrobial defences or evolved specific compounds to cope with a much narrower range of pathogenic threats.

## References

[1] Stow A, Briscoe D, Gillings M, Holley M, Smith S (2007). Antimicrobial defences increase with sociality in bees..

[2] Evans JD, Spivak M (2010). Socialized medicine: Individual and communal disease barriers in honey bees.. J Invert Pathol.

[3] Stow A, Beattie A (2008). Chemical and genetic defenses against disease in insect societies.. Brain Behav Immun.

[4] Turnbull C, Hoggard S, Gillings M, Palmer C, Stow A (2010). Antimicrobial strength increases with group size: implications for social evolution..

[5] Turillazzi S, Mastrobuoni G, Dani FR, Moneti G, Pieraccini G (2006). Dominulin A and B: Two new antibacterial peptides identified on the cuticle and in the venom of the social paper wasp *Polistes dominulus* using MALDI-TOF, MALDI-TOF/TOF, and ESI-Ion trap.. J Am Soc Mass Spectrom.

[6] Brothers DJ (1999). Phylogeny and evolution of wasps, ants and bees (Hymenoptera, Chrysidoidea, Vespoidea and Apoidea).. Zool Scripta.

[7] Hunt JH (2007). The evolution of social wasps..

[8] Smith SM, Beattie AJ, Gillings MR, Holley MP, Stow AJ (2008). An enhanced miniaturized assay for antimicrobial prospecting.. J Microbiol Meth.

[9] Pilgrim EM, von Dohlen CD, Pitts JP (2008). Molecular phylogenetics of Vespoidea indicate paraphyly of the superfamily and novel relationships of it component families and subfamilies.. Zool Scripta.

[10] Evans HE, O'Neil KM (2007). The sand wasps: Natural history and behaviour..

[11] Wcislo WT, Cane JH (1996). Floral resource utilisation by solitary bees (Hymenoptera: Apoidea) and exploitation of their stored food by natural enemies.. Annu Rev Entomol.

[12] Brockmann HJ (1980). Diversity in the nesting behaviour of mud-daubers (*Trypoxylon politum* Say; Sphecidae).. Fla Entomol.

[13] Cooper KW (1957). Biology of Eumeninae wasps. V. Digital communication in wasps.. J Exp Zool.

[14] Evans HE, Matthews RW (1973). Behavioural observations on some Australian spider wasps (Hymenoptera: Pompilidae).. T Roy Ent Soc London.

[15] Inoue M, Endo T (2008). Below-ground host location by *Campsomeriella annulata* (Hymenoptera: Scoliidae), a parasitoid of scarabaeid grubs.. J Ethol.

[16] Kloos WE (1980). Natural populations of the genus *Staphylococcus*.. Annu Rev Microbiol.

[17] R Development Core Team (2010). http://www.R-project.org/.

[18] Sunnucks P, Hales DF (1996). Numerous transposed sequences of mitochondrial cytochrome oxidase I-II in aphids of the genus Sitobion (Hemiptera: Aphididae).. Mol Biol Evol.

[19] Dowton M, Austin AD (1998). Phylogenetic relationships among the microgastrid wasps (Hymenoptera: Braconidae): Combined analysis of of 16S and 28S rDNA genes and morphological data.. Mol Phylogenet Evol.

[20] Folmer O, Black M, Hoeh W, Lutz R, Vrijenhoek R (1994). DNA primers for amplification of mitochondrial cytochrome c oxidase subunit I from diverse metazoan invertebrates.. Mol Mar Biol Biotech.

[21] Tamura K, Dudley J, Nei M, Kumar S (2007). MEGA4: Molecular Evolutionary Genetics Analysis (MEGA) software version 4.0.. Mol Biol Evol.

[22] Swofford DL (2003). PAUP*. Phylogenetic Analysis Using Parsimony (*and Other Methods)..

[23] Drummond AJ, Rambaut A (2007). BEAST: Bayesian evolutionary analysis by sampling trees.. BMC Evol Biol.

[24] Posada D (2008). jModelTest: Phylogenetic Model Averaging.. Mol Biol Evol.

[25] Rambaut A, Drummond AJ (2007). http://beast.bio.ed.ac.uk/Tracer.

[26] Rambaut A (2009). http://tree.bio.ed.ac.uk/software/figtree/.

[27] Hines HM, Hunt JH, O'Connor TK, Gillespie JJ, Cameron SA (2007). Multigene phylogeny reveals eusociality evolved twice in vespid wasps.. PNAS.

